# Limited-Memory Fast Gradient Descent Method for Graph Regularized Nonnegative Matrix Factorization

**DOI:** 10.1371/journal.pone.0077162

**Published:** 2013-10-21

**Authors:** Naiyang Guan, Lei Wei, Zhigang Luo, Dacheng Tao

**Affiliations:** 1 National Key Laboratory of Parallel and Distributed Processing, School of Computer Science, National University of Defense Technology, Changsha, Hunan, China; 2 Centre for Quantum Computation and Intelligent Systems and the Faculty of Engineering and Information Technology, University of Technology, Sydney, Australia; Rensselaer Polytechnic Institute, United States of America

## Abstract

Graph regularized nonnegative matrix factorization (GNMF) decomposes a nonnegative data matrix 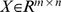 to the product of two lower-rank nonnegative factor matrices, i.e., 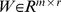 and 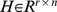 (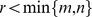) and aims to preserve the local geometric structure of the dataset by minimizing squared Euclidean distance or Kullback-Leibler (KL) divergence between *X* and *WH*. The multiplicative update rule (MUR) is usually applied to optimize GNMF, but it suffers from the drawback of slow-convergence because it intrinsically advances one step along the rescaled negative gradient direction with a non-optimal step size. Recently, a multiple step-sizes fast gradient descent (MFGD) method has been proposed for optimizing NMF which accelerates MUR by searching the optimal step-size along the rescaled negative gradient direction with Newton's method. However, the computational cost of MFGD is high because 1) the high-dimensional Hessian matrix is dense and costs too much memory; and 2) the Hessian inverse operator and its multiplication with gradient cost too much time. To overcome these deficiencies of MFGD, we propose an efficient limited-memory FGD (L-FGD) method for optimizing GNMF. In particular, we apply the limited-memory BFGS (L-BFGS) method to directly approximate the multiplication of the inverse Hessian and the gradient for searching the optimal step size in MFGD. The preliminary results on real-world datasets show that L-FGD is more efficient than both MFGD and MUR. To evaluate the effectiveness of L-FGD, we validate its clustering performance for optimizing KL-divergence based GNMF on two popular face image datasets including ORL and PIE and two text corpora including Reuters and TDT2. The experimental results confirm the effectiveness of L-FGD by comparing it with the representative GNMF solvers.

## Introduction

NMF factorizes a given nonnegative data matrix 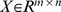 into two lower-rank nonnegative factor matrices, i.e., 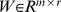 and 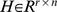, where 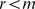 and 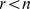. It is a powerful dimension reduction method and has been widely used in many fields such as data mining [1] and bioinformatics [2]. Since NMF does not explicitly guarantee parts-based representation [3], Hoyer [4] proposed sparseness constrained NMF (NMFsc) which incorporates the sparseness constraint into NMF. To utilize the discriminative information in a dataset, Zafeiriou *et al*. [5] proposed discriminant NMF (DNMF) to incorporate Fisher's criteria in NMF for classification. Sandler and Lindenbaum [6] proposed an earth mover's distance metric-based NMF (EMD-NMF) to model the distortion of images for image segmentation and texture classification. Guan *et al*. [7] investigated Manhattan NMF (MahNMF) for low-rank and sparse matrix factorization of a nonnegative matrix and developed an efficient algorithm to solve MahNMF.

Since NMF and its extensions do not consider geometric structure of a dataset, they perform unsatisfactorily in some tasks such as clustering. To consider the local geometric structure of a dataset in NMF, Cai *et al.* [8] proposed graph regularized nonnegative matrix factorization (GNMF) which encodes the geometric structure in a nearest neighbor (NN) graph for data representation. Along this direction, Guan *et al*. [9] extended GNMF to manifold-regularized discriminative NMF (MD-NMF) to incorporate discriminative information in a dataset by using margin maximization. The same authors proposed a nonnegative patch alignment framework (NPAF) [10] to unify such NMF-based nonlinear dimension reduction methods. Because the objective functions of GNMF and NPAF are jointly non-convex with respect to both factor matrices, their optimizations are difficult.

Similar to NMF, GNMF is NP-hard. It is impossible to obtain its global minimum in polynomial time [11]. Fortunately, GNMF is convex with respect to each factor matrix, i.e., the sub-problems for updating individual factor matrix are convex, and thus it can be solved by recursively updating both factor matrices in the frame of block coordinate descent. Cai *et al*. [8] exploited the multiplicative update rule (MUR) to update each factor matrix alternately until convergence to a local minimum. MUR searches one step along the rescaled negative gradient direction with a step size setting to one. Since the step size is non-optimal, MUR does not sufficiently utilize the convexity of the sub-problems of GNMF. Although both [12] and [13] can solve squared Euclidean distance based NMF efficiently, they are not general enough to optimize Kullback-Leibler (KL) divergence based GNMF. Recently, Guan *et al*. [9] proposed a fast gradient descent (FGD) method to accelerate MUR for KL-divergence based GNMF. FGD searches the optimal step size along the rescaled negative gradient direction by using Newton's method. Since FGD sets a single step size for the whole factor matrix, it has the risk of shrinking to MUR, i.e., the final step size shrinks to one. To overcome this deficiency, Guan *et al*. [10] further proposed a multiple step-size FGD (MFGD) method which sets a step size for each row of *W* and each column of *H*, and searches the optimal step size vector by using the multivariate Newton's method. MFGD converges more rapidly than FGD, but the dimensionalities of the Hessian matrices used in the line search procedures for updating both factor matrices are too high, i.e., the Hessian matrices are *m*×*m*-dimensional and *n*×*n*-dimensional for optimizing *W* and *H*, respectively. Therefore, MFGD suffers from the following two drawbacks: 1) both the Hessian inverse operators and their multiplications with the corresponding gradients cost too much computational time, and 2) the dense Hessian matrices consume too much memory.

To overcome the aforementioned deficiencies of MFGD, motivated by limited memory BFGS (L-BFGS) [14], we propose a limited-memory FGD (L-FGD) method to directly approximate the multiplication of the Hessian inverse and the gradient for the multivariate Newton method in MFGD. Since L-BFGS stores only a few most recent historical gradients, L-FGD greatly reduces the memory cost compared to MFGD which stores the Hessian matrix. In addition, since L-BFGS converges as fast as the multivariate Newton method and avoids calculating the Hessian inverse, L-FGD converges in similar iteration rounds and costs much less CPU time in each iteration round. Therefore, L-FGD is much more efficient than MFGD both in terms of memory complexity and time complexity. The theoretical analysis and experimental results on real-world datasets including two popular face image datasets, i.e., ORL [15] and PIE [16], and two text corpora, i.e., Reuters [17] and TDT2 [18] show that L-FGD converges much more rapidly than MUR, FGD, and MFGD. Furthermore, we apply the L-FGD method to solve KL-divergence based GNMF and confirm its effectiveness by evaluating its clustering performance. Experimental results on two popular face image datasets, i.e., ORL [15] and PIE [16], confirm the effectiveness of L-FGD compared with the representative GNMF solvers.

The remainder of this paper is organized as follows: Section II briefly reviews GNMF and its optimization algorithms; Section III presents the L-FGD method; Section IV evaluates its efficiency and effectiveness by experiments; and Section V concludes this paper.

## Analysis

This section reviews several significant works on nonnegative matrix factorization (NMF) including sparseness constrained NMF (NMFsc, [4]), earth mover's distance metric-based NMF (EMD-NMF, [6]), discriminant NMF (DNMF, [5]), and graph regularized NMF (GNMF, [8]).

### A. NMF

Given a nonnegative data matrix 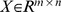, NMF [1] aims to find two lower-rank nonnegative matrices 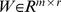 and 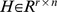 by minimizing the following objective
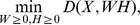
where 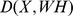 measures the distance between *X* and *WH*, which is usually the squared Euclidean distance, i.e., 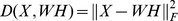 or the Kullback-Leibler (KL) divergence:
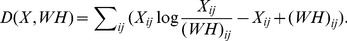
(1)


### B. NMFsc

It is well-known that NMF does not guarantee parts-based representation of data [3]. To remedy this problem, Hoyer [4] proposed to explicitly constrain the sparseness of each column of *W* and each row of *H*, i.e.,
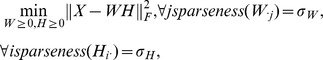
where 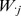 and 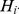 stand for the *j*-th column of *W* and the *i*-th row of *H*, respectively, and 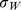 and 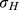 are two constants in 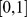. The sparseness of a vector 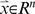 is defined as
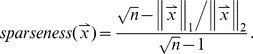



### C. EMD-NMF

Since both Euclidean distance and KL-divergence cannot appropriately qualify the errors in images or histograms, the standard NMF does not perform well in image analytics. To make NMF more appropriate for image analytics, Sandler and Lindenbaum [6] proposed earth mover's distance (EMD) metric-based NMF (EMD-NMF) because EMD qualifies the errors in images or histograms better than other metrics. The objective of EMD-NMF is
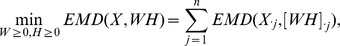
where EMD between any two same sized matrices equals to the summation of EMD distances between their column vectors. Please refer to [6] for more details about EMD.

Although NMF, NMFsc, and EMD-NMF perform well in some tasks, they completely ignore the discriminative information of the dataset, and thus perform unsatisfactorily in some pattern recognition tasks.

### D. DNMF

To utilize the labels of the dataset, Zaferiou *et al*. [5] proposed discriminant NMF (DNMF) to incorporate Fisher's criterion in NMF, i.e.,
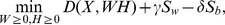
where 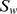 and 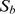 are within-class scatter and between-class scatter of *H*, respectively. Since NMF itself does not assume data points are Gaussian distributed, it is improper to use Fisher's criterion to retain the discriminative information for subsequent classification.

### E. GNMF

Graph regularized nonnegative matrix factorization (GNMF) [8] encodes the geometric structure of the dataset based on manifold regularization [19] and sheds a light to overcome the deficiency of DNMF. It constructs an adjacent graph, i.e., *G*, for a dataset and keeps the neighbor relationship of nodes on *G* during projecting data points from the high-dimensional space to the low-dimensional subspace, i.e.,
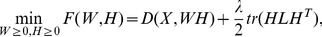
(2)where 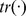 signifies the trace operator, *L* is the graph Laplacian of *G*, and *λ* is a positive tradeoff parameter. Since GNMF utilizes the intrinsic geometric information, it has discriminating power and performs well in clustering.

Since GNMF is jointly non-convex with respect to both *W* and *H*, its optimization is quite difficult. Although some efficient solvers of NMF, e.g., NeNMF [12], can be utilized to optimize the squared Euclidean distance based GNMF, they are not general enough to optimize the KL-divergence based GNMF. In the following section, we will introduce a new efficient solver for KL-divergence based GNMF.

## Results

This section first revisits the existing GNMF solvers, i.e., multiplicative update rule (MUR), fast gradient descent (FGD), and multiple step-sizes FGD, and then introduces limited-memory FGD algorithm.

### A. GNMF Solvers Revisit

Multiplicative update rule (MUR) is one of the most popular algorithms for optimizing GNMF. According to [9], the MUR for KL-divergence based GNMF is
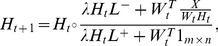
(3)and
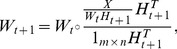
(4)where 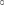 signifies the element-wise multiplication operator and *t* signifies the iteration counter, *L* ^+^ and *L* ^−^ can be obtained with 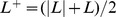, 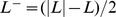. Both *L* ^+^ and *L* ^−^ are nonnegative symmetric matrices because *L* is a symmetric matrix.

Although (3) and (4) reduce the objective function of (2), they converge slowly because MUR is intrinsically a rescale gradient descent method with a step size equal to 1. To accelerate MUR, Guan *et al*. [9] proposed fast gradient descent (FGD) which sets a step-size for each factor matrix (*W* or *H*) and searches the optimal step size along the rescaled negative gradient direction in each iteration round. Taking the procedure of updating *H* as an example, the objective function of searching the optimal step-size is
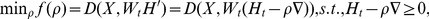
(5)where 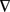 is the rescaled negative gradient calculated as follows:
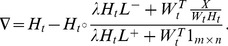
(6)


**Figure 1:**
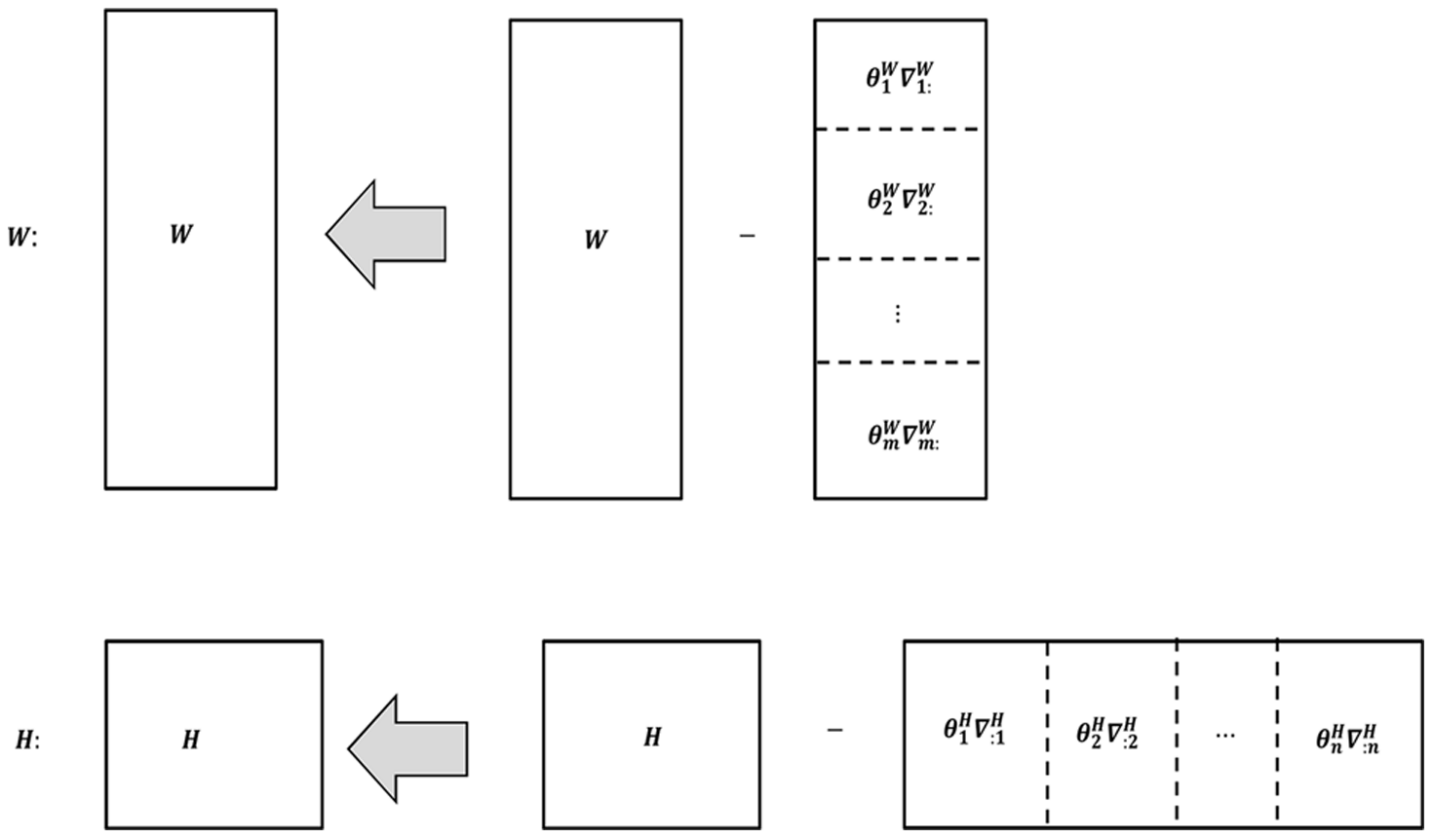
Descent of both *W* and *H* along their rescaled negative gradient directions in MFGD.

Since the objective function of (5) is convex, it can be solved by using Newton's method without increasing the computational cost. Although FGD greatly accelerates MUR, it risks shrinking MUR because the incorporated constraint may result in *ρ* = 1. To remedy this problem of FGD, multiple step-sizes FGD (MFGD, [10]) considers the step-size for each row of *W* and each column of *H*. Thus it is necessary to calculate a vector 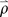 for each matrix in each iteration round. Figure 1 shows the step-size assignment of *W* and *H* in MFGD.

It is clear that the objective of searching the optimal step size vector for *H* is
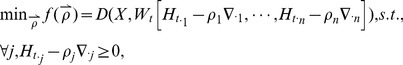
(7)where 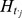 is the *j*-th column of 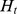 and 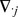 is the *j*-th column of 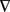. Since the constraints are incorporated on columns of *H* and 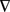, MFGD reduces the risk of shrinking to MUR and thus accelerates MUR in most cases. Since problem (7) is convex, we can employ the multivariate Newton's method to obtain the optimal solution. However, the Hessian matrix used in MFGD has high dimensionality and thus MFGD has two additional disadvantages: 1) it costs too much memory especially when *m* or *n* is large, and 2) the Hessian inverse operator and its multiplication with gradient are computationally too expensive.

### B. Limited-memory FGD

**Figure 2:**
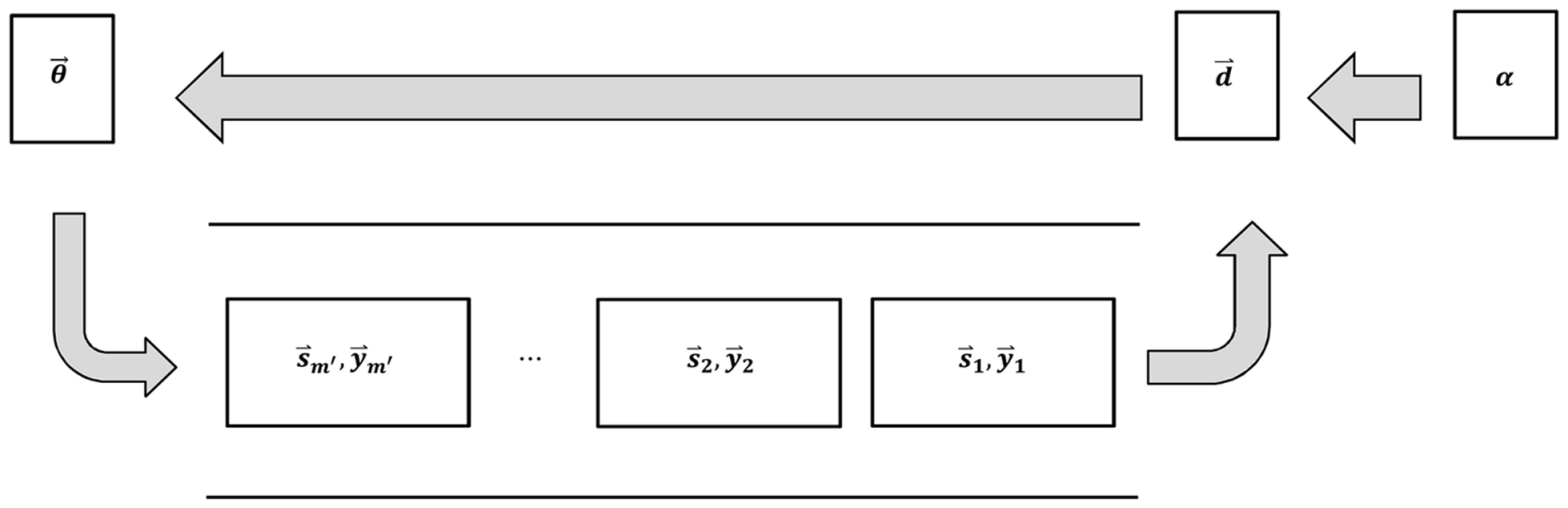
Basic process of L-FGD.

Motivated by L-BFGS [20], we directly approximate the multiplication of the Hessian inverse and gradient to overcome the deficiencies of MFGD. L-BFGS uses historical information to approximate the Hessian inverse, thus avoiding the complex matrix inverse operator. For efficiently solving our line search problem (7), we develop a limited-memory FGD (L-FGD) method. The updating rule of L-FGD is given by
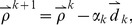
(8)where *k* signifies the iteration counter of the line search, 
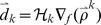
 is the multiplication of Hessian inverse 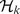 and gradient 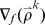 of 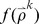, and 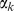 is the step-size. According to [14], L-FGD approximates the Hessian inverse by using a recursion process, i.e.,
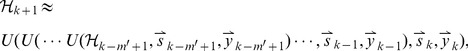
(9)where 
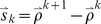
, 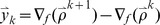, and 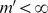. The recursion function is defined as follows:
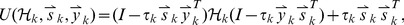
(10)where 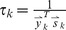. From (9) and (10), L-FGD utilizes finite recent pairs 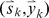 to approximate the Hessian inverse and refreshes the set of pairs iteratively by replacing the oldest pair with the newest pair as showed in Figure 2. Due to the recursion process in (9), L-FGD avoids the Hessian inverse operator and thus costs much less CPU time than MFGD.

However, the recursion process (9) retains an approximate Hessian inverse matrix and thus L-FGD consumes too much memory. To overcome this deficiency, we utilize the two loop recursion process [14] to directly approximate the multiplication of Hessian inverse and gradient in two steps summarized in **Algorithm 1** (See Table 1). Similar to (9), 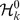 represents an approximation of the Hessian inverse, however, it can be set to a scaled identity matrix, i.e., 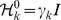, where 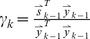. In this case, line 6 can be directly calculated and thus L-FGD avoids storing the Hessian inverse matrix. This strategy greatly reduces the memory costs of L-FGD. To search the optimal 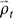 along the rescaled negative gradient of *H* for solving (7), we initialize 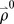 and update it by using the L-BFGS method until the criterion 
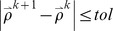
 is met, where *tol* is a predefined tolerance. Similar to MFGD, the update formula of L-FGD is
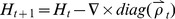
(11)where 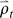 is the obtained optimal step-size vector. Since L-FGD needs to fill the queue of 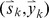 pairs, we set two initial points 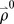 and 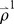 in each call of **Algorithm 1** to avoid null pairs in the first iterations. The procedure of L-FGD for updating 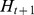 is summarized in **Algorithm 2** (See Table 2).

**Table 1:** Summary of the two loop recursion algorithm for L-FGD.

**Algorithm 1.** Two loop recursion procedure for L-FGD
**Input**: Pairs 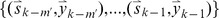 , Gradient 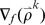 .
**Output:** 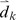
1. Initialize 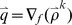 .
2. **For** 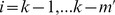
3. Compute 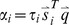 .
4. Update 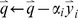 .
5. **End For**
6. Initialize 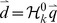 .
7. **For** 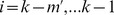
8. Compute 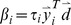 .
9. Update 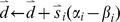
10. **End For**
11. 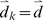

**Table 2:** Summary of the proposed limited memory fast gradient descent algorithm.

**Algorithm 2.** Limited Memory FGD (L-FGD)
**Input**: 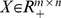 , *L*, 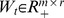 , 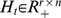
**Output**: 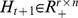
1. 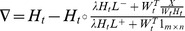 .
2. Initialize 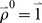 , 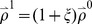 , 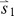 , 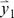 , 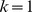 .
3. Calculate relative gradient 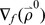 and 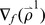 .
**Repeat**
4. Update 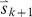 and 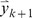 .
5. Calculate 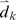 by using **Algorithm 1**.
6. Update 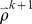 : 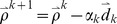 .
7. Calculate 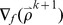 at 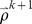 .
8. Update 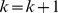 .
**Until** {Stopping when the criterion 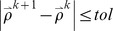 is met.}
9. Set 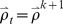 and calculate 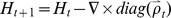 .

In line 2 of **Algorithm 2**, 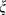 is a small positive constant that regularizes the speed of convergence, e.g., 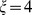 on dense dataset and 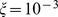 on sparse dataset, and *tol* is the predefined tolerance, e.g., 10^−3^. In line 6 of **Algorithm 2**, 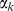 is the step size of the *k*-th iteration round, e.g., 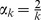 in our experiment. The main time cost is spent on lines 1 and 5, whose time complexities are 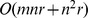
and 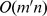, respectively. Thus its total complexity is 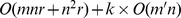, where *k* stands for the total number of iterations of **Algorithm 2**. Since the L-BFGS method converges as rapidly as the multivariate Newton method, *k* is usually small, and the time cost of one iteration of L-FGD is comparable to that of MUR, i.e., 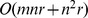. However, L-FGD converges much more quickly than MUR in terms of number of iterations because the used step-size is optimal, thus the overall time cost of L-FGD is much less than that of MUR. In Table 3, we compare the time and memory complexities of L-FGD with those of MUR, FGD and MFGD.

**Table 3:** The time and memory complexity of one iteration round of MUR, FGD, MFGD and L-FGD for GNMF.

Algorithm	Time Complexity	Memory Complexity
MUR	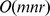	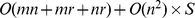
FGD	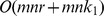	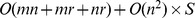
MFGD	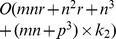	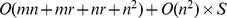
L-FGD	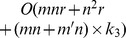	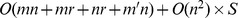

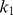
, 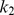 and 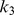: iteration number of Newton's method, multivariate Newton method, and L-BFGS methods for line search in FGD, MFGD, and L-FGD, respectively; S: sparsity of the alignment matrix; p<n.

The second column of Table 3 compares the time complexities of one iteration of MUR, FGD, MFGD, and L-FGD and shows that L-FGD takes much less time than MFGD because it avoids calculating the Hessian inverse. Although L-FGD has similar time complexity to MUR, it accelerates MUR in each iteration round and costs much less overall time complexity. By comparison with FGD, it reduces the risk of shrinking to MUR. The third column of Table 3 compares the memory complexity of four methods, where the term 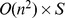 is caused by the graph Laplacian matrix, which is usually sparse. The promising advantage of L-FGD is that it greatly reduces the memory cost of MFGD and is thus much more suitable for large-scale datasets.

## Experiments

In this section, we evaluate the efficiency of L-FGD for solving GNMF by comparing it with MUR [9], FGD [9] and MFGD [10] on ORL [15] and PIE [16] face image datasets and Reuters [17] and TDT2 [18] text corpora. We implement all algorithms in MATLAB program on a workstation which contains a 3.4GHz Intel (R) Core (TM) processor and an 8GB RAM. We use the 0–1 weighting scheme for constructing a k-nearest neighbor graph in GNMF. For fairness of comparison, all algorithms start from an identical initial point. To evaluate the efficiency of L-FGD for GNMF, we stop all GNMF solvers until they reach an identical objective value. To this end, we first use MUR [9] to optimize the KL-divergence of GNMF and stop when the following condition is satisfied with precision *ε* = 10^−4^:
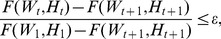
(12)where 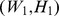 is the initial point and both matrices are set to random dense matrices. We then use three other methods to optimize the function and stop when each reaches the same objective value of MUR. Meanwhile we count the number of iterations and time cost to compare their efficiency. To evaluate the effectiveness of L-FGD for GNMF, we test the clustering performance obtained by these GNMF solvers. Taking the same measure as that of efficiency, we calculate and compare their normalized mutual Information and accuracy. Each experiment is repeated 20 times to avoid the impact of randomness.

The ORL dataset [15] includes 400 images collected from 40 individuals. Each individual has 10 images and each image is cropped into 32×32 pixels and reshaped into a 1024-dimensional long vector. The PIE dataset [16] contains 11,554 pictures collected from 68 individuals with varying poses and illuminations. In this experiment, we select all the images taken at Pose 27 of each individual to construct a subset containing 1428 images. Each image is also cropped into 32×32 pixels and reshaped to a 1024-dimensional vector.

The Reuter corpus [17] contains 21578 documents which compose of 135 categories. We discard those documents belonging to multiple categories and the obtained dataset contains 8293 documents in 65 categories. The TDT2 corpus [18] consists of 11201 on-topic documents which are categorized into 96 groups. We remove the documents appearing in two or more categories and obtain 9394 documents in 30 categories.

### A. Study of Efficiency

**Figure 3:**
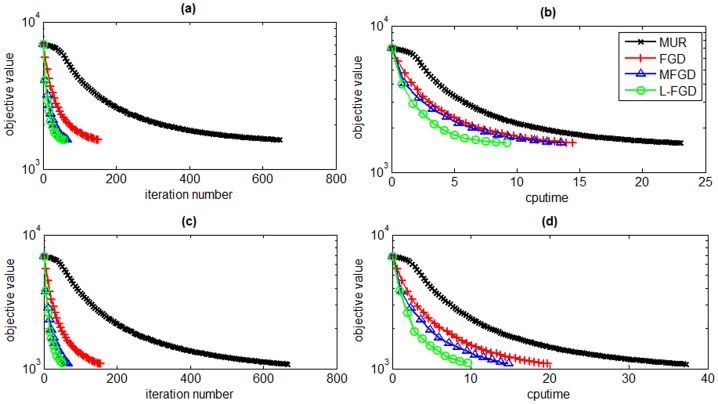
Objective values versus number of iterations and CPU time on the ORL dataset. The reduced dimensionality is set to 50 (a and b) and 100 (c and d).

**Figure 4:**
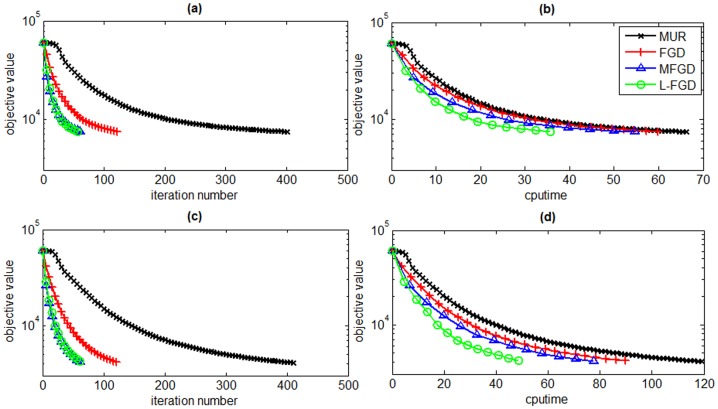
Objective values versus number of iterations and CPU time on the PIE dataset. The reduced dimensionality is set to 50 (a and b) and 100 (c and d).

In this section, we evaluate the efficiency of L-FGD for solving GNMF by comparing it with MUR [9], FGD [9] and MFGD [10]. The sizes of the data matrices for ORL and PIE datasets are 400×1024 and 1428×1024, respectively. The subspace dimensionality is set to 50 and 100 to study the scalability of L-FGD. The tradeoff parameter *λ* is set to 0.001 and the number of nearest neighbors is set to 5. Figures 3 and 4 present the iteration numbers and time cost of the four algorithms on the ORL and PIE datasets, respectively.

Figures 3 and 4 show that L-FGD spends the least CPU time among all GNMF solvers to reach the same objective value. The number of iterations of L-FGD is almost the same with MFGD, but L-FGD greatly reduces the time of calculating the inverse Hessian matrix in MFGD. Although L-FGD searches multiple step sizes in each iteration round like MFGD, its total CPU time is less than that of FGD. Since the step size of MUR equals 1, its time cost is the highest.

**Figure 5:**
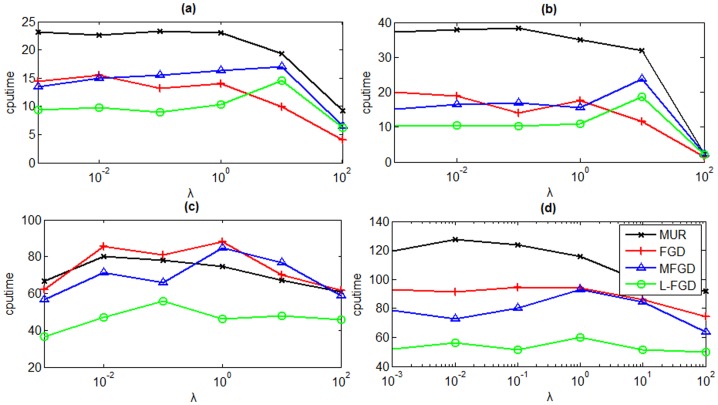
CPU time versus parameter *λ* on both ORL (a and b) and PIE (c and d) datasets. The reduced dimensionality is set to 50 (a and c) and 100 (b and d).

The GNMF (2) has two essential parameters, including the number of nearest neighbors *k* and the tradeoff parameter *λ*. The latter has great effect on the speed of convergence. Figure 5 shows the performance of algorithms on ORL and PIE respectively when *λ* is searched on the grid {0.001, 0.01, 0.1, 1, 10, 100}. It shows that L-FGD costs less CPU time than MUR, FGD, and MFGD in most cases on the ORL dataset and converges most rapidly on the PIE dataset.

**Figure 6:**
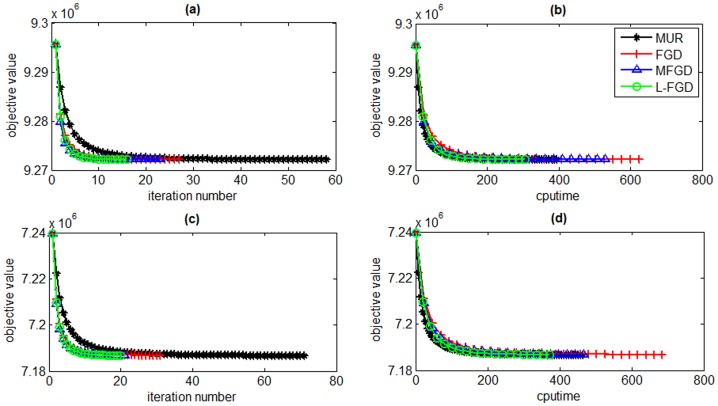
Objective values versus number of iterations and CPU time on the Reuters dataset. The subspace dimensionality is set to 100 (a and b) and 500 (c and d).

**Figure 7:**
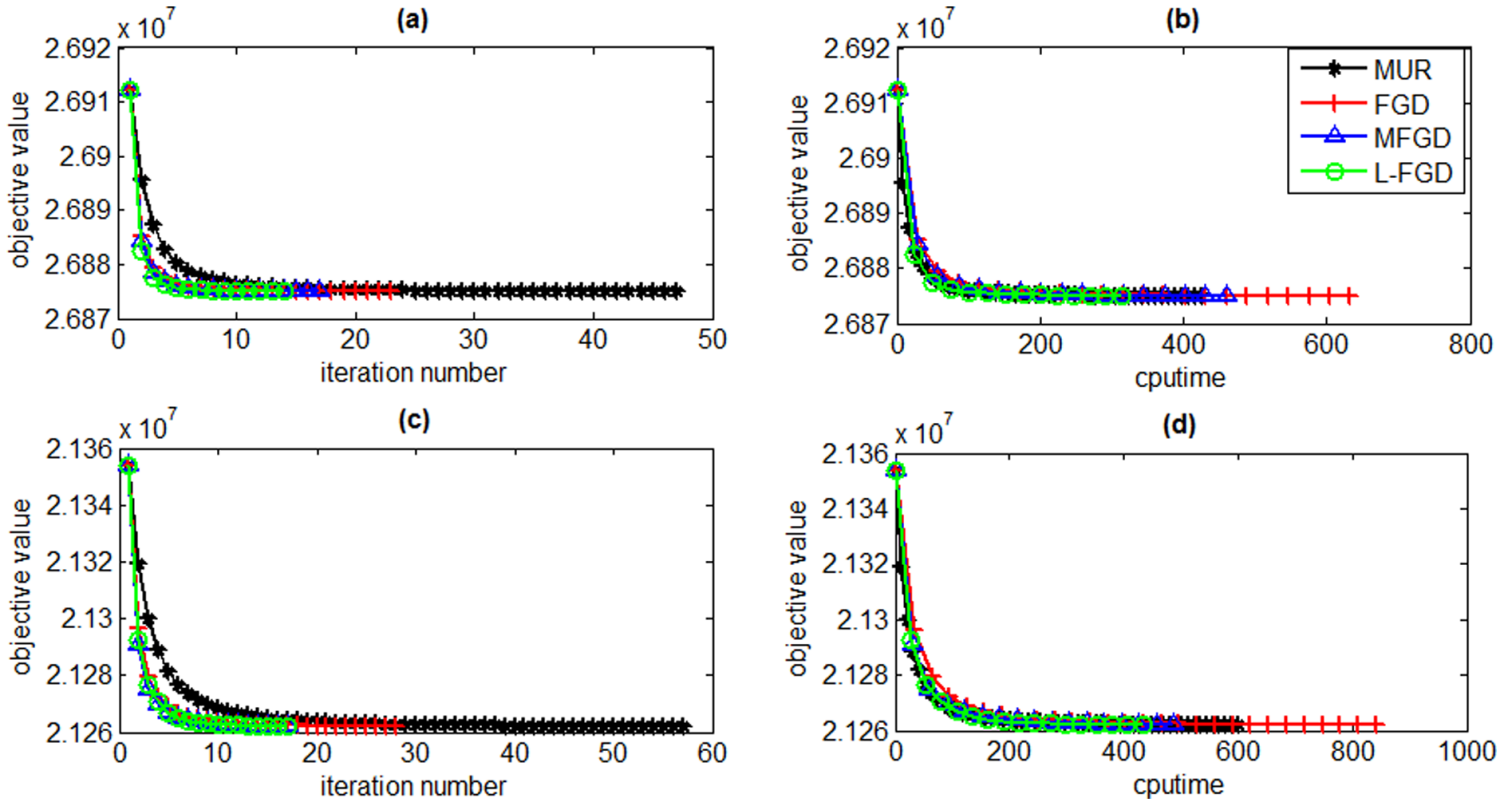
Objective values versus number of iterations and CPU time on the TDT2 dataset. The subspace dimensionality is set to 100 (a and b) and 500 (c and d).

In order to validate the proposed L-FGD algorithm on medium scale datasets, we compare it with other GNMF solvers, i.e., MUR, FGD, and MFGD, on two document corpora including Reuters and TDT2. The dimensionalities of Reuters and TDT2 are 8293×18933 and 9394×36771, respectively. We select the first 15000 columns of TDT2 matrix for our evaluation due to the memory limit of our test platform. The subspace dimensionality is respectively set to 100 and 500 to study the scalability of L-FGD. The tradeoff parameter *λ* is set to 0.001 and the number of nearest neighbors is set to 5. Figures 6 and 7 present the objective values versus iteration numbers and CPU time of L-FGD, MUR, FGD, and MFGD on both Reuters and TDT2 datasets, respectively. They depict that the proposed L-FGD algorithm converges much faster than MUR, FGD, and MFGD on both Reuters and TDT2 datasets.

In summary, L-FGD optimizes GNMF with quite light computational burden and rather limited memory cost, and thus makes it possible to extend GNMF to various practical problems such as supervised learning [21] [22] and tensor factorization [23] [24] on medium scale datasets.

### B. Study of Clustering Performance

In this section, we test the effectiveness of L-FGD for solving GNMF by comparing its clustering performance with those of MUR, FGD and MFGD. We randomly selected *K* class samples from the ORL and PIE datasets to perform K-means on the results of GNMF to obtain both the clustering accuracy and normalized mutual information. The cluster number *K* varies from 2 to 10. For each *K*, 20 tests run on each randomly chosen cluster to avoid the impact of randomness. Table 4 and Table 5 show the mean and standard error of the accuracy and normalized mutual information on the ORL and PIE dataset, respectively.

**Table 4:** Normalized mutual information and accuracy of GNMF solved by MUR, FGD, MFGD, and L-FGD on the ORL dataset.

*K*	Normalized Mutual Information (%)				Accuracy (%)			
	MUR	FGD	MFGD	L-FGD	MUR	FGD	MFGD	L-FGD
2	64.3±41.7	63.8±41.8	63.8±41.8	64.9±41.1	86.3±16.7	86.5±16.1	86.5±16.1	87.0±16.0
3	69.1±25.1	70.6±24.0	70.6±24.0	70.6±24.0	81.7±16.5	81.8±16.8	81.8±16.8	81.8±16.8
4	70.2±18.0	71.4±18.1	71.6±17.4	71.0±18.3	77.9±15.6	77.6±16.8	77.5±17.0	78.6±15.7
5	71.7±11.1	71.8±11.1	71.1±11.4	71.8±11.1	76.2±11.3	76.0±11.2	75.8±11.2	76.0±11.2
6	70.3±11.7	68.3±11.5	69.8±11.4	69.2±11.4	73.9±10.4	72.8±9.5	73.3±10.4	73.1±10.0
7	75.6±6.6	75.9±6.8	75.1±7.9	75.1±7.0	73.6±8.5	74.4±9.1	73.5±10.5	73.9±9.4
8	72.5±10.9	73.7±9.7	72.9±10.9	73.5±9.3	69.8±12.1	71.7±11.6	71.3±12.8	71.6±10.9
9	71.5±5.7	72.8±5.7	72.6±6.0	72.8±5.6	67.7±7.8	68.9±8.4	69.1±7.9	68.6±8.0
10	74.6±6.4	73.7±6.8	74.0±6.0	74.7±6.5	69.8±9.0	68.8±9.1	69.1±8.3	69.2±8.4
Avg.	71.1±15.2	71.3±15.1	71.3±15.2	71.5±14.9	75.2±12.0	75.4±12.1	75.3±12.3	75.6±11.8

**Table 5:** Normalized mutual information and accuracy of GNMF solved by MUR, FGD, MFGD, and L-FGD on the PIE dataset.

*K*	Normalized Mutual Information (%)				Accuracy (%)			
	MUR	FGD	MFGD	L-FGD	MUR	FGD	MFGD	L-FGD
2	75.5±33.6	83.8±32.0	84.4±32.5	75.2±36.5	91.6±13.2	93.8±14.1	93.7±14.8	90.8±15.4
3	96.0±8.3	96.9±7.6	96.9±7.6	96.9±7.6	98.2±3.8	98.6±3.5	98.6±3.5	98.6±3.5
4	95.7±8.2	97.6±4.9	98.0±4.5	90.5±8.5	96.4±8.1	98.7±2.8	98.9±2.6	93.2±6.5
5	98.3±4.6	98.8±4.1	98.8±4.1	98.5±4.4	98.1±6.3	98.5±5.8	98.5±5.8	98.4±5.8
6	96.2±5.4	97.1±5.0	96.8±5.0	96.6±5.0	96.5±5.9	97.8±4.2	97.6±4.2	97.4±4.2
7	94.9±5.5	94.0±6.0	93.5±6.0	93.9±5.9	93.8±7.5	91.6±8.7	90.8±8.6	91.6±8.7
8	93.9±3.6	93.9±4.3	93.6±4.6	93.5±4.2	91.4±6.8	90.2±8.0	89.4±8.2	89.5±7.9
9	93.8±4.2	93.1±3.6	93.1±3.6	93.7±3.9	90.6±7.0	87.9±6.4	87.9±6.2	89.7±6.7
10	92.1±2.9	92.1±3.6	91.8±3.6	92.0±3.4	85.6±6.9	85.8±6.6	84.7±7.1	85.6±7.4
Avg.	92.9±8.5	94.1±7.9	94.1±7.9	92.3±8.8	93.6±7.3	93.7±6.7	93.3±6.8	92.8±7.3

Tables 4 and 5 show that the four GNMF solvers have nearly the same normalized mutual information and accuracy whatever the cluster number *K* is. In summary, the proposed L-FGD method accelerates MFGD while keeping the clustering performance of GNMF.

## Conclusions

Motivated by L-BFGS, this paper presents a new method L-FGD to accelerate the MFGD algorithm for GNMF. Since the memory cost of MFGD storing the Hessian matrix is high, and much time is taken to calculate its inverse in the used multi-variable Newton method, it is both memory-consuming and time-consuming. L-FGD needs nearly the same iteration rounds as MFGD before convergence but greatly reduces the time costs needed by each iteration round. Experiment results show that L-FGD converges much more rapidly than MFGD in terms of CPU time and retains the effectiveness of the solution obtained for GNMF.
