# Correction: Limited-Memory Fast Gradient Descent Method for Graph Regularized Nonnegative Matrix Factorization

**DOI:** 10.1371/annotation/96c3a19d-e19d-4241-b819-0c1fc0282f9b

**Published:** 2014-01-06

**Authors:** Naiyang Guan, Lei Wei, Zhigang Luo, Dacheng Tao

Affiliation number 1 for the first, second, and third authors is incorrect. The correct affiliation is: National Laboratory for Parallel and Distributed Processing, School of Computer Science, National University of Defense Technology, Changsha, Hunan, China. 

